# What Are We Measuring? Evaluating Physician-Specific Satisfaction Scores Between Emergency Departments

**DOI:** 10.5811/westjem.2019.4.41040

**Published:** 2019-04-26

**Authors:** Brian Sharp, Jordan Johnson, Azita G. Hamedani, Emilia B Hakes, Brian W. Patterson

**Affiliations:** University of Wisconsin School of Medicine and Public Health, BerbeeWalsh Department of Emergency Medicine, Madison, Wisconsin

## Abstract

**Introduction:**

Most emergency departments (ED) use patient experience surveys (i.e., Press Ganey) that include specific physician assessment fields. Our ED group currently staffs two EDs – one at a large, tertiary-care hospital, and the other at a small, affiliated, community site. Both are staffed by the same physicians. The goals of this study were to determine whether Press Ganey ED satisfaction scores for emergency physicians working at two different sites were consistent between sites, and to identify factors contributing to any variation.

**Methods:**

We conducted a retrospective study of patients seen at either ED between September 2015 and March 2016 who returned a Press Ganey satisfaction survey. We compiled a database linking the patient visit with his or her responses on a 1–5 scale to questions that included “overall rating of emergency room care” and five physician-specific questions. Operational metrics including time to room, time to physician, overall length of stay, labs received, prescriptions received, demographic data, and the attending physician were also linked. We averaged scores for physicians staffing both EDs and compared them between sites using t-tests. Multiple logistic regression was used to determine the impact of visit-specific metrics on survey scores.

**Results:**

A total of 1,012 ED patients met the inclusion criteria (site 1=457; site 2=555). The overall rating-of-care metric was significantly lower at the tertiary-care hospital ED compared to our lower volume ED (4.30 vs 4.65). The same trend was observed when the five doctor-specific metrics were summed (22.06 vs 23.32). Factors that correlated with higher scores included arrival-to-first-attending time (p=0.013) and arrival-to-ED-departure time (p=0.038), both of which were longer at the tertiary-care hospital ED.

**Conclusion:**

Press Ganey satisfaction scores for the same group of emergency physicians varied significantly between sites. This suggests that these scores are more dependent on site-specific factors, such as wait times, than a true representation of the quality of care provided by the physician.

## INTRODUCTION

Under the Affordable Care Act, increasing emphasis has been placed on delivery of healthcare that is both patient-centered and high quality with the aim of incentivizing better value and outcomes.^1^,^2^ While an improved patient experience likely contributes to improved quality of care and outcomes, measurement of this facet of quality is difficult to accomplish.^3^,^4^ Currently, this measurement typically involves patient survey scores assessing both the overall experience and specific aspects of the emergency department (ED) visit, including a physician-specific section. Increasingly, payers are using these scores to modify provider reimbursement.^5^

Numerous studies conducted in the ED have demonstrated the many factors that influence patients’ satisfaction with their visits. While good communication, attitude and interpersonal skills demonstrated by ED staff are associated with increased patient satisfaction scores, factors such as wait time, patient demographics and acuity, as well as crowding, also influence scores.^6^–^20^ Some studies have even suggested that higher patient satisfaction scores are tied to more drug prescriptions and advanced imaging.^3^,^4^,^21^

Regarding physician-specific metrics, Bendesky et al. in 2016 showed that patient satisfaction scores differed for emergency physicians (EP) based on the setting in which they were practicing. Specifically, satisfaction scores were consistently lower in an ED setting when compared to an urgent care. This finding suggests that even metrics that attempt to narrowly assess the patient-provider relationship are subject to external factors.^22^ Given that urgent cares have been found to be viewed favorably in terms of quality and value among patients, further study is needed to control for site-specific effects on patient satisfaction.^23^

In August 2015 our health system opened a second ED at a university-affiliated site that is staffed by the same emergency medicine faculty group. There are some operational differences between the sites, including consultant availability as well as the level of involvement of residents and advanced practice providers (APP) in care. However, most ancillary services offered are largely identical, including radiology studies (radiograph, computed tomography, ultrasound, magnetic resonance imaging) and lab services. This presents an ideal scenario to compare physician-specific Press Ganey ratings. Our objective was to evaluate consistency of physician-specific patient satisfaction scores between the two sites.

## METHODS

This was a retrospective cohort study examining Press Ganey surveys at two different EDs. Site 1 is situated in a suburban area, has inpatient medicine services with limited subspecialty services available. It is approximately 12 miles from site 2 and has an annual ED volume of 11,221 (during the study period). Site 2 is an academic, tertiary-care hospital in an urban environment with an annual ED volume of 55,561 (during the study period). Both EDs are staffed by board-certified or board-eligible EPs. In addition to EP staffing, site 1 (smaller, suburban site) had limited APP staffing (four hours of coverage daily) during the study period, whereas site 2 (academic center) had significant resident and APP staffing with their involvement in most patients’ care.

Discharged patients from both EDs received a survey (via mail or email) administered by Press Ganey Associates (South Bend, Indiana). We included in the analysis patients cared for by EPs who worked at both sites from September 2015–May 2016, a period chosen based on availability of data for analysis. Further requirements included a minimum of 10 evaluations per site per physician (which had the effect of limiting inclusion to full-time physicians with significant practice at both sites) and full survey responses. Returned surveys were linked to the encounter so that treating physician, demographics, date and time of visit, vital signs, and any tests performed could be obtained. We excluded from the analysis patients who were cared for by more than one EP within a visit.

**Population Health Research Capsule**

What do we already know about this issue?

*Physician-specific scores on patient satisfaction surveys are often used as a proxy for the quality of care delivered by emergency physicians*.

What was the research question?

Do patient satisfaction scores differ for the same physicians staffing two different emergency departments?

What was the major finding of the study?

*Patient satisfaction scores for the same physicians were lower at the higher volume/longer wait time site*.

How does this improve population health?

*Press Ganey scores, intended to measure patient satisfaction with physicians, may be more influenced by site-specific than physician-specific factors*.

We used patient responses to physician-specific questions. These questions included the following: overall rating of care; courtesy of the doctors who cared for you; degree to which these doctors took the time to listen to you; concern these doctors showed to keep you informed about your treatment; concern these doctors showed for your comfort while treating you; and degree to which these doctors advocated for your care. Possible ratings ranged from 1 (very poor) to 5 (very good). Additional variables were selected based on potential impact on patient experience based on prior literature; these included age, race, gender, acuity, means of arrival, time interval from arrival to rooming, time interval from arrival to leaving the ED, and whether patients received any labs or advanced imaging.^7^–^14^,^16^,^19^,^20^

We obtained data from the electronic health record (EHR), which exists in one continuous instance at both sites. Press Ganey data were linked to EHR data reports by departmental staff during the creation of the dataset. We analyzed data using Stata 15 (Statacorp, College Station, Texas). We compared demographic and Press Ganey data using t-test for continuous data and chi^2^ test for categorical data. To evaluate physician-specific metrics, we evaluated the response rate for overall rating of care as well as the sum of the five physician-specific metrics. A logistic regression model was created to evaluate the impact of site and physician on scores while controlling for covariates. Given the high proportion of returned surveys with a total score of 25 (highest rating across all scores), we dichotomized outputs into scores of 25 vs all other scores for the regression analysis. Additionally, we ranked all included physicians from highest to lowest in Press Ganey scores at both sites. Given our sample size, we expected to detect a difference in mean score of 0.18 from the mean Press Ganey scores at site 2 (the academic site) with a power level of 0.8 at an alpha of 0.05 based on a two-tailed test.

## RESULTS

### Characteristics of Study Subjects and Sites

After applying exclusion criteria, we included 1012 encounters in the analysis: 457 from site 1 and 555 from site 2. The Figure details patient attribution by site. Thirteen EPs met the minimum of 10 returned surveys per site and were included in the analysis. By physician, the median number of surveys returned at site 1 was 29 (range 10–82, interquartile ratio [IQR] 17–41); at site 2 the number returned was 37 (range 29–72, IQR 30–52).

Patient demographics were similar between sites, including age, race, gender, and mode of arrival (Table 1). Wait times differed between the two sites, with shorter arrival-to-room and arrival-to-discharge times observed at site 1. At site 1 the mean arrival-to-first attending time was 18.0 minutes (standard deviation [SD] 19.9) and the arrival-to-ED-departure time was 200.5 minutes (SD 101.0) compared to 75.8 minutes (SD 66.1) and 254.8 (126.3) respectively at site 2.

### Main Results

A total of 13 EPs (48% of full-time, non-pediatric providers) met the minimum of 10 returned surveys per site and were included in the analysis. By physician, the median number of surveys returned was 29 at site 1 (range 10–82, IQR 17–41) and 37 at site 2 (range 29–72, IQR 30–52). Mean Press Ganey satisfaction scores for provider overall rating of care were higher at site 1 compared to site 2 (Table 2). The same trend was seen for the sum of the five physician-specific metrics, which included the following: courtesy of the doctors who cared for you; degree to which these doctors took the time to listen to you; concern these doctors showed to keep you informed about your treatment; concern these doctors showed for your comfort while treating you; and degree to which these doctors advocated for your care.

In the regression analysis, no individual physician was associated with a significant odds ratio for achieving or not achieving high Press Ganey scores. Being seen at site 1 and shorter arrival-to-room and arrival-to-discharge times were associated with a higher incidence of high scores. Patient-specific factors such as age, race, gender, arrival mode, and acuity were not associated with differences in scores, nor were any individual physicians associated with statistically significant increases or decreases in scores. The regression model had a c-statistic of 0.68 and a non-significant Hosmer-Lemeshow goodness of fit test at 0.278 (Table 3). When ranking physicians between sites (Table 4), we observed no discernible correlation between the two sets of rankings.

## LIMITATIONS

This study was conducted within one health system and trends may differ in other organizations. Additionally, while both sites are EDs with similar patient populations, one difference of note is that EPs who staff site 2 typically work with resident physicians and APPs, including physician assistants and nurse practitioners, which is less common at site 1. Differences in physician-specific-scores may be due to the fact that physicians at site 2 were rated along with their residents and APPs. While we would argue that this is one of the site-specific characteristics of site 2, with regard to this site it is important to note that the effect of residents or APPs overall was not directly measured and may be a major driver of the effect observed.

The study was also limited by its retrospective design. Due to the methodology of data collection (reporting from EHR records) it is possible that physicians were incorrectly matched to patient encounters in some cases, although this is unlikely as all cases with more than one assigned physician were dropped from analysis. In our setting, as has been reported in institutions elsewhere, Press Ganey survey response rates were low. While this is a common feature of Press Ganey data in general, we cannot extrapolate our results to other scenarios in which response rates were higher, in which case physician-specific ratings may be more accurate and less dependent on external factors as observed here.

## DISCUSSION

This study compared physician-specific patient satisfaction scores for EPs who practice in two different EDs. We observed that Press Ganey survey scores were consistently lower for the same physicians practicing at site 2 compared to site 1. This is similar to the findings of Bendesky et al. (2016), who found that patient satisfaction scores of the same EPs differed based on the site where they were practicing.^22^ Our results further support that even provider-specific patient satisfaction scores are strongly correlated with site-specific factors such as time spent waiting for a room and total length of the stay. This is also consistent with prior studies that demonstrate shorter wait times are associated with increased patient satisfaction.^8^–^10^

While other investigators have found associations between satisfaction scores and factors such as patient age, race, acuity, and arrival mode, our analysis did not show any of these associations.^7^–^9^, ^11^,^19^ Notably, our predominantly Caucasian patient population may imply that other ethnicities were under-represented to the extent that no difference in satisfaction could be detected. Additionally, other factors that could have impacted the physician-specific metric score difference include physician time spent with patients and the level of involvement of residents and APPs in care.

A physician’s Press Ganey score is increasingly being used as a proxy for the quality of care they provide. While we feel that improved patient experience scores are a worthy goal for EPs given the multiple benefits that have been shown to correlate with an improved patient experience (compliance, decreased likelihood of malpractice lawsuits, etc),^6^,^7^,^17^,^24^ our results further bring into question whether currently used patient- experience ratings are an accurate measurement of this. Further study is needed to control for site-specific factors to better isolate the provider-patient relationship before these ratings can be used in a meaningful way. Until then, our results suggest the need to use caution when interpreting provider-specific satisfaction scores, especially when these scores are linked to things such as financial incentives and promotion or tenure.

## CONCLUSION

We found that Press Ganey scores for the same group of physicians differed between two sites. Scores were higher at the lower-volume site where wait times were shorter. These results suggest that Press Ganey scores are affected by factors outside of the physician’s control. Scores should be interpreted with caution, especially when used as a proxy for the quality of care provided by the physician.

## Figures and Tables

**Figure:**
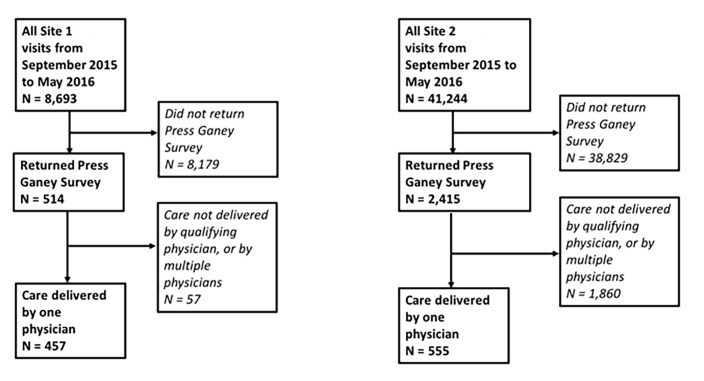
Patient attribution by site.

**Table 1:** Respondent demographics.

	Site 1		Site 2	

Demographic Variable	Number	% (95% CI)	Number	% (95% CI)
				
Total responses	457		555	
Mean age (SD)	53.8 (15.7)		53.4 (17.7)	
p=0.702				
Race				
White	442	96.7 (94.6–98.0)	509	91.7 (89.1–93.7)
Other	15	3.3 (2.0–5.4)	46	8.3 (6.3–10.9)
Gender				
Male	170	37.2 (32.9–41.7)	194	34.9 (31.1–39.0)
Female	287	62.8 (58.3–67.1)	361	65.1 (61.0–68.9)
Mode of arrival				
Self/family/friends	398	87.1 (83.7–89.9)	480	86.5 (83.4–89.1)
EMS/police	59	12.9 (10.1–16.3)	75	13.5 (10.9–16.6)
Acuity				
2	76	16.6 (13.5–20.3)	130	23.4 (20.1–27.1)
3	302	66.1 (61.6–70.3)	342	61.6 (57.5–65.6)
4	76	16.6 (13.5–20.3)	81	14.6 (11.9–17.8)
5	3	0.7 (0.2–2.0)	1	0.4 (0.1–1.4)

*CI*; confidence interval; *SD*, standard deviation; *EMS*, emergency medical services.

**Table 2:** Press Ganey satisfaction scores at both sites.

	Site 1		Site 2		P value

Survey item	Mean score	95% CI	Mean score	95% CI	
					
Overall satisfaction with visit	4.65	4.58–4.72	4.30	4.21–4.39	<0.001
Sum of five other physician-specific scores	23.32	22.98–23.63	22.06	21.70–22.44	<0.001

*CI*, confidence interval.

**Table 3:** Regression analysis of factors affecting the “overall rating of care” score.

Metric	Odds ratio	P value	95% CI
Patient age	1.018	<0.001	1.009–1.027
Race (vs. white)			
American Indian or Alaska Native	0.738	0.746	0.117–4.638
Asian	1.444	0.463	0.541–3.858
Black	0.637	0.273	0.284–1.427
Unknown	0.721	0.655	0.171–3.038
Patient gender (vs. male)			
Female	0.885	0.399	0.666–1.176
Site (vs. Site 2)			
Site 1	0.594	0.003	0.421–0.838
Acuity (vs. 2)			
3	0.880	0.471	0.623–1.245
4	0.821	0.421	0.508–1.327
5	2.301	0.474	0.236–22.467
Arrival (vs. self/family/friends)			
EMS/police	0.736	0.137	0.491–1.103
Physician (vs. 1)			
2	0.622	0.152	0.325–1.191
3	0.705	0.282	0.372–1.334
4	0.531	0.076	0.264–1.068
5	0.775	0.513	0.362–1.661
6	0.727	0.328	0.383–1.378
7	0.975	0.938	0.510–1.864
8	0.647	0.221	0.323–1.298
9	1.717	0.191	0.764–3.856
10	1.139	0.693	0.597–2.171
11	0.583	0.096	0.309–1.101
12	0.723	0.412	0.333–1.570
13	0.843	0.635	0.417–1.705
Arrival-to-first-attending time	0.996	0.013	0.994–0.999
Arrival-to-ED-departure time	0.999	0.038	0.997–1.000

*CI*, confidence interval; *EMS*, emergency medical services; *ED*, emergency department.

**Table 4:** Physician rankings by site based on mean of physician-specific Press ganey scores.

Rank	Site 1	Site 2
1	I	E
2	B	A
3	H	I
4	J	J
5	L	D
6	A	C
7	M	B
8	F	G
9	E	K
10	C	M
11	K	F
12	D	H
13	G	L
